# Central Attention and a Dual Path Convolutional Neural Network in Real-World Tree Species Recognition

**DOI:** 10.3390/ijerph18030961

**Published:** 2021-01-22

**Authors:** Yi Chung, Chih-Ang Chou, Chih-Yang Li

**Affiliations:** 1 College of Human Development and Health, National Taipei University of Nursing and Health Sciences, Taipei 11219, Taiwan; 2 Xin Ji International Company, New Taipei 234014, Taiwan; catchsob@gmail.com; 3 Department of Computer Science and Information Engineering, National Taiwan University, Taipei 10617, Taiwan; taipingeric@gmail.com

**Keywords:** plant recognition, deep learning, dual path convolutional neural network, visual attention, mobile application

## Abstract

Identifying plants is not only the job of professionals, but also useful or essential for the plant lover and the general public. Although deep learning approaches for plant recognition are promising, driven by the success of convolutional neural networks (CNN), their performances are still far from the requirements of an in-field scenario. First, we propose a central attention concept that helps focus on the target instead of backgrounds in the image for tree species recognition. It could prevent model training from confused vision by establishing a dual path CNN deep learning framework, in which the central attention model combined with the CNN model based on InceptionV3 were employed to automatically extract the features. These two models were then learned together with a shared classification layer. Experimental results assessed the effectiveness of our proposed approach which outperformed each uni-path alone, and existing methods in the whole plant recognition system. Additionally, we created our own tree image database where each photo contained a wealth of information on the entire tree instead of an individual plant organ. Lastly, we developed a prototype system of an online/offline available tree species identification working on a consumer mobile platform that can identify the tree species not only by image recognition, but also detection and classification in real-time remotely.

## 1. Introduction

### 1.1. Motivation

Plants are important resources on Earth to maintain ecosystems, promote the medical sector, and increase agricultural productivity and sustainability [1]. Moreover, regional biodiversity studies, population size estimates of endangered species, and species distribution due to climate change depend on the accuracy of plant identification [2].

One of the most obvious features of organic life is its remarkable diversity [3]. Despite the variation of organisms, a more experienced eye soon distinguishes that organisms can be grouped into taxa [4]. These days, with the continuous loss of biodiversity [5], the demand for routine species identification has risen; meanwhile, the number of experienced experts is limited and reduced [6].

Nowadays, the growth of technology and urbanization has decreased the knowledge and uses of plants by humans. However, the environment and the natural resources raise a growing concern. An accurate automated identification system enables non-experts with only limited botanical training and expertise to contribute to the survey of the world’s biodiversity [4], thus providing inexperienced people, who had no knowledge in botany, with a tool to recognize the plants that surround them would be a great advance [7].

Plant identification is not only the work of botanists and ecologists, but also useful or essential for large segments of society, from professionals to the general public. However, the identification of plants by conventional ways is difficult, time consuming, and frustrating for novices due to the use of specific botanical terms [4].

Recently, computer science research, especially image processing and pattern recognition techniques, have been proposed to plant taxonomy in order to eventually overcome the deficiency in human identification abilities [4]. Field researchers, land managers, civil servants, educators, and the interested public would greatly benefit from up-to-date and accessible tools automating the process of species identification. Notably, relevant technologies, such as mobile devices, digital cameras, and remote access to databases, are ubiquitously available, accompanied by significant advances in computer vision [4]. With the popularity of smartphone devices, web-based and stand-alone applications have been developed to help users identify plant species [8].

### 1.2. Deep Learning

Deep learning approaches have shown promising results in various computer vision problems, including the plant identification task driven by the success of convolutional neural networks (CNN) [4]. The essence of deep learning is its capacity to create and extrapolate new features from raw representations of input data without having to be told explicitly which features to use and how to extract them [9].

The latest studies on plant identification utilize these techniques and achieve significant improvements over methods developed in the decade before. However, a few of them were conducted in controlled environments, with lighting and background being constants that limited the flexibility of user experiences [4].

### 1.3. Challenges in Real-World Scenarios

The training dataset is the cornerstone, upon which deep learning algorithms depend [10]. There were some plant species benchmark datasets, most of which were leaf images scanned or photographed with plain background such as Swedish leaf [11], Flavia [12], Leafsnap [13], and Intelligent Computing Laboratory (ICL) [14]. The Plant Cross Language Evaluation Forum (PlantCLEF) [15] dataset contained more realistic images, such as multiple organs like fruits, flowers, leaves, and stems in the natural environment. However, the collected species were mainly focused on the Guiana shield and the Northern Amazon rainforest, and the average number of image per species was three times less than the previous edition of PlantCLEF; some species may even contain only one image.

In fact, most botanists sometimes simultaneously observe several organs of the same plant in order to disambiguate species easily confused when observing only a single organ [16]. A plant classification method is presented using a combination of leaf, flower, and bark photos of the same tree species, and achieves recognition accuracy on multi-organ better than the accuracy on a single organ [17]. Rather than a full view of the entire plant, a single organ photograph could lead to the high risk of recognition errors because of disproportion to the real world. Additionally, these previous bench datasets were limited in the number of species due to the tremendous effort for collecting them, especially for biodiversity in regional differences. Plant recognition is still challenging due to the great variability of these characteristics within images from the same species and also the similarity of some characteristics between different species [18].

Here, we proposed a comprehensive study of deep CNNs to extract the feature vectors from the images of entire plant observations.

### 1.4. Central Attention

The background of photos taken in real-world ways is usually complicated. A real-world plant image contains more than one object, i.e., target plants and other interferents in the background. Furthermore, target plants are possibly touching or overlapped with the background objects. However, the salient objects what we pay attention to are to be recognized in an image. For an image, human visual attention allows us to rapidly locate the most important information in a scene [9], and the most useful point is focused on with our attention at first sight for a given object [10].

In addition to the interference of the non-target, there are also non-valuable redundancy. The object is recognized only with the sketchy and concentrated screenage or information born in our mind although there is a large amount of content. Other non-salient parts are ignored or neglected. Therefore, we are not even aware of the redundancy during the first judgement [19].

Xiao et al. proposed a deep learning framework with attention cropping [19]. The input images were cropped in terms of visual attention before recognized. Attention cropping can focus on the real interesting target and remove the interferences, and was accomplished with the generated saliency map using the saliency detection approach [20]. Results show that the targets and foregrounds were reserved and the distant surroundings and redundancies were tailored out after attention cropping. The final identification was made using pre-trained deep CNNs. The results of CNNs using attention cropping augmentation were superior to those of CNNs without attention cropping. InceptionV3 with attention cropping outperformed classic InceptionV3 by 4.2% accuracy.

Visual attention prediction aims to predict scene locations where a human observer may fixate [21]. Recently, driven by the success of deep learning in object recognition, many deep learning based attention models [22,23,24,25] were proposed, and generally gave impressive results. The attention-based approaches [26,27,28] focus on preserving the main subject or visually important area in the scene after cropping [21]. These methods usually place the crop window over the most visually significant regions according to certain attention scores [29,30,31,32].

The most important information and what we are most interested are facilitated to be located using attention cropping to fulfill real-world identification although there are different scenarios and the background is complex in realistic images. Notably, attention cropping possesses greater advantage and better performance for real-world recognition compared with the conventional recognition where the background is simple [19]. Furthermore, when taking pictures, users tend to place the subject in the middle according to our observation and survey.

Based on this attention concept and the user experience observation, we defined the salient objects which are to be recognized in an image are focused in the center. We cropped the image from the center, acquired it as the input of one CNN path, and named the operation central attention.

### 1.5. Dual-Path CNN

Several dual path CNN models were developed in multiple domains, and generally showed remarkable results [33,34,35,36,37]. In the present study, we constructed a dual path CNN, one of which is the central attention path while the other is a classic CNN path. The backbone architecture of the former path is a classic CNN path with inputs from the central attention cropping images while the later one is a classic CNN path with inputs from the original images.

### 1.6. Mobile Application

With the popularization of intelligent mobile devices, the automated plant identification technology on the edge side plays a crucial role in the fields of ecological environment supervision, ecological science popularization, and foreign plant invasion monitoring [38].

A smartphone consists of everything required for the implementation of a mobile plant identification system, including a camera, a processor, a user interface, and an Internet connection. These requirements make smartphones particularly suitable for field use by professionals and the general public. However, these devices still have less storage capacity, available memory, network bandwidth, and computational power than desktop computers or servers, limiting algorithmic choices. Due to these limitations, it can be tempting to offload some of the processing to a high-performance server that requires a reliable Internet connection. Using an online service can be attractive when dataset or algorithm is likely to be updated regularly or when they have large computational and memory requirements. However, in remote areas where plant identification applications are likely to be most useful, an Internet connection may be unavailable or unreliable. The alternative approach is to use efficient algorithms that run directly on the device without the need for a network connection or a support server but with potential limitations in the classification performance [39,40].

Xingse [41], a mobile application for plant recognition developed in China, has not documented several endemic plant species in Taiwan even with similar environmental conditions, such as *Millettia pinnata*, not to mention some of the well-known mobile apps [42,43,44] that were sampled outside of Asia may result in insufficient plant species in their database due to regional differences.

Numerus automatic systems were designed for plant identification, but only a few mobile applications can be used simply to identify tree species just by clicking on a button slightly or moving around the mobile camera with real-time object detection and classification. Up to now, the latest developments about the automatic identification system was presented. However, these studies were mostly limited to leaf images [45]. To solve these problems, we aim to develop a tree recognition mobile application with a superior user experience which is simpler, intuitive, and much more accurate.

### 1.7. The Aim of the Study

We aim to introduce the central attention concept implemented with a dual path CNN for real-world tree species recognition. For application, the purpose of this study is to provide the general public with better user experiences in recognizing tree species, so as to reduce barriers to entry for the ecological filed, while offering the plant lover a seamless user experience to assist one in identifying confusing tree species and improving the recognition efficiency.

To begin with, we collect our own dataset containing 14 common tree species in Taiwan with the images of entire plant observations by taking pictures in the field. Then, we introduce a central attention concept which is constructed in a dual path deep learning framework based on the CNN, trained the model by using the self-collected dataset, and underwent the test of identifying the trees based on their morphological features. Furthermore, in order to verify the validity of the proposed framework, we used a uni-path traditional CNN and a uni-path central attention method to perform the same experiment for comparative analysis. Experiments showed that our proposed dual path deep learning framework had achieved good effect of identification. Finally, we implemented the tree species recognition into an Android mobile application, which can detect object with mobile cameras in real-time, display the label, and overlay on the camera image. No Internet connection is required for using this app.

### 1.8. Related Works

#### 1.8.1. Deep learning

Deep learning related works were summarized in Table 1. Different modern and advanced models have been proposed for automated plant recognition as the deep learning technology advances [1].

One of the first studies on plant identification using a CNN is Lee et al’s leaf classifier which utilized the AlexNet model pre-learned on the ImageNet Large Scale Visual Recognition Challenge 2012 (ILSVRC2012) dataset and reported an average accuracy of 99.7% on the MalayaKew (MK) leaf dataset consisting of 44 species [9,46]. TreeID, a plant species identification system, was developed with a simple three-layer CNN [47]. Wu et al. used a four-layer CNN for classification of leaves accompanied by the Parametric Rectified Linear Unit (PReLU) activation function instead of traditional Rectified Linear Unit (ReLU), and were able to obtain an accuracy of 94.8% on the ICL dataset with 50 different species of leaves [48].

Bao et al. compared a traditional shallow architecture, which extracted a feature histogram of an oriented gradients (HOG) vector along with an Support Vector Machine (SVM) classifier, to a deep five-layer CNN on the Flavia and Swedish leaf datasets. The deep architecture presented greater accuracy, and worked well on classification problem of leaves based on the shape of veins [49]. Zhang et al. utilized a seven-layer CNN to classify the Flavia dataset, and reached 94.69% accuracy [50]. Barre et al. further improved this result by using a 17-layer CNN and obtained an accuracy of 97.9% validated on the LeafSnap, Flavia, and Foliage datasets [51].

Zhu et al. employed a 19-layer CNN in combination with a linear classifier SVM, and outperformed conventional methods based on hand-crafted features [45]. Pearline et al. utilized Visual Geometry Group 16 (VGG 16) CNN architecture with logistic regression (LR) that resulted in an accuracy of 97.14% for Leaf12 dataset, while the VGG 19 CNN architecture with a logistic regression classifier reached an accuracy of 96.53%, 96.25% and 99.41% on Folio, Flavia, and Swedish leaf datasets, respectively. Moreover, they found that VGG 16 or 19 deep learning architectures with an LR classifier resulted in higher accuracy compared with InceptionV3 and Inception-ResNet-v2. [52]. However, InceptionV4 and Inception-ResNet-v2 CNN models won the ExpertLifeCLEF Plant Identification Task 2018 and LifeCLEF Plant Identification Task 2019 [15,54].

Sun et al. studied the ResNet architecture, which solves the vanishing gradient and degradation problems [55]. They found a 26-layer network to achieve best performance with 99.65% on the Flavia dataset with a simple background, and 91.78% on Beijing Forestry University 100 (BJFU100) dataset, which consists of 100 species of ornamental plants multiplied by 100 photos in natural scenes [53]. Eventually, Bodhwani et al. designed a 50-layer deep residual learning framework, and achieved a recognition rate of 93.09% with 0.24% error on the LeafSnap dataset [1]. In spite of intensive and elaborate studies on automated plant species identification, only a little research resulted in approaches which can be used by the general public [4].

#### 1.8.2. Mobile Applications

Mobile Applications related works were summarized in Table 2. LeafView [56], a tablet PC-based application, can be used offline for the automated identification of species directly in the field. A user took a picture of a single leaf on a blank background, and the app extracted the shape feature and matched it with the existing species. The system then showed the top matches in a few seconds with text descriptions and additional photographs, or marked unknown for further study. The system returned the correct species in the top ten matches 90–97% of the time.

Leafsnap [42] is the first widely distributed Android classification application. Implemented as a mobile app, it used computer vision techniques for identifying tree species from photographs of their leaves on a simple background. Based on leaf contour features, the app utilized integral measure to compute functions of the curvature at the boundary over multiple scales for classification [64], and retrieved leaf images similar to the query by nearest neighbors (NN) for identification [46]. Then, it was up to the user to make the final decision on what species matched the unknown one. LeafSnap achieved a top-1 recognition rate of 73% and a top-5 recognition rate of 96.8% for 184 tree species [65]. The app had attracted a considerable number of downloads, but also received many critical reviews [66] because it fails to handle cluttered backgrounds and does not pay enough attention to both large intra-species visual variations and strong inter-species visual similarities which are typical in the botanical domain [4]. Although efficient for a single input, its processing time hindered the classification of larger sets with a rate of 5.4 s per sample [33]. Moreover, it can make the identification only with access to the Internet, the query image must be taken by the local camera, and is restricted to tree species of the Northeastern United States [39].

Pl@ntNet [43] is an image sharing and retrieval application for botanical identification, available on three front-ends, an iOS app, an Android app, and a web interface. Each allowed users to submit one or several images of a plant with the intention of obtaining a list of the most likely species. Pl@ntNet adopted deep learning techniques for image classification pre-trained on the ImageNet dataset and periodically fine-tuned on steadily growing Pl@ntNet data since 2015 [67]. The application has been downloaded by more than 5 million users worldwide. Joly et al. [68] evaluated the application which supported the identification of 2200 species at that time, and reported a 69% top-5 identification rate for single images [4].

FOLIA [57], an interactive iOS application, helps to identify a plant species in the natural environment. In order to perform this function, the software first lets the user photograph an unrecognized plant with a smartphone camera. It then extracts high-level morphological characteristics to determine the list of the best matches.

ApLeafis [58] is an Android-based plant leaf identification system in JAVA based on content-based image retrieval technique. An isolated leaf needs to be properly shot on a light, plain, and uniform background. In contrast to Leafsnap, ApLeafis can function without an Internet connection, and select digital images as query images.

Similar to Leafsnap, a leaf based plant identification system for Android used a client-server implementation was developed. To begin with, a leaf photo was taken with the phone, and then being sent to the server for analysis in order to identify the species. Furthermore, the server processed two steps. First was to check if the uploaded photo was a leaf. Then, if the image was validated containing a leaf, the species identification would be activated; otherwise the system would ask for another photo. The identification method was based on Speeded Up Robust Features combined with Bag of Words (BOW) and supervised learning. Finally, the species information would be displayed to the user. The results showed that the method obtained the 95.94% average accuracy [59].

A low computational approach was proposed with an offline mobile application for Android using OpenCV [61]. Leaf images were captured with the device’s camera and must have a uniform background in order to simplify the segmentation. The shape and color features were extracted. The algorithm calculated the geometric feature and then polar Fourier transform, and trained using k-nearest neighbor (k-NN) classifier. After that, two nearest classes were selected on the basic of smallest distance which was further rectified by the color features using a decision tree. The classification process was done on the mobile device itself.

In another Android application in Java, classification can either be done on the server for more computationally expensive algorithms or offline on the device [40]. The online mode involved sending only the feature vector to the server rather than the actual image. The feature extraction was done on the phone itself, significantly reducing bandwidth requirements for the server connection that allowed the most consistent and reliable match speed and continued to function without a network connection, for example, in the wild. Finally, the server returned a dynamic webpage in the device’s browser with the closest matches to the database. For the offline recognition mode of the algorithm, the feature extraction, database search and extraction of the top 10 results took between 145–171 ms. The database consisted of 300 KB of features, and 19 MB of images (or 1.5 MB if only a single image per class was used for display).

An Android client application, which interacts with a leaf recognition algorithm running on the server through a Simple Object Access Protocol-based web service, was developed [62]. The developers used the Scale Invariant Feature Transform (SIFT) algorithm combining with BOW model for feature vector dimensionality reduction, and Support Vector Machine (SVM) classifier. SIFT features were used because they are invariant in scale, rotation, camera viewpoint, and illumination. The BOW model could reduce the high dimensionality of the data space. SVM has a simple structure, comparatively fast speed on training, and it is easy to implement. OpenCV was utilized for the actual image processing. The system was trained to classify 20 species and obtained 96.48% accuracy level.

ApLeaf [63], an Android-based plant leaf identification system, was developed to automatically identify plant species by the photographs of tree leaves. To start with, given a query image taken by the camera or already existed in the local database, the authors segmented it into a binary image by threshold segmentation, and moved the stem by the Tophat method at the preprocessing step. The image should be a single leaf placed on an untextured and light background without other clutter. Next, important features, such as pyramid histograms of oriented gradients and color, were extracted and fused to form the final feature space. Finally, they used the histogram intersection to predict the class and returned to the users the top species which match the query image best. The system was trained on the ImageCLEF2012 Plant Identification database which contains 126 tree species from the French Mediterranean area. It depended on several aspects in computer vision, including segmenting leaves from background, extracting various features by distance measure for species identification, showing users the list of matched species. The performance of the app showed up to 90% accuracy.

Other similar smartphone applications served as identification aides and/or as repositories of educational information were publicly available [69], such as PlantSnap [70], WildSnap [71], Forest Tree Identification [72], PictureThis—Plant Identifier (the English version of Xingse) [73], About My Woods [74], and Southeast Early Detection Network (SEEDN) [75].

Through deep learning, an iOS mobile application for automated recognition of plants and flowers was introduced [10]. Unlike other apps which focus on static pictures for feature classification, they used video data that compensates for the possibly lost information when comparing a static image with many others images of plants and flowers. This application reported the capability to identify 122/125 plants and 47/50 genera selected with degrees of confidence up to 95%. They also describe the performance speed up through the use of Cloud-based resources.

iNaturalist [44], automatically identified animals and plants at the species level, was launched for Android and iOS by iNaturalist.org in 2017. At first, the app only offered crowd-sourced species identification. Users posted a picture of a plant or animal, and a community of scientists and naturalists identified it. A taxon was raised to “research grade” as soon as more than 2/3 of the involved identifiers agreed in their identification of an observation. It used NVIDIA GPUs (NVIDIA Corporation, Santa Clara, CA, USA) and the Compute Unified Device Architecture (CUDA) deep neural network library along with the TensorFlow deep learning framework. That provided training of the neural networks with an image database which have been labeled by the site’s community of experts [76]. iNaturalist already identified over 10,000 different species with a new species added to the model every 1.7 h [77].

Although numerous progresses have been made in plant identification research, several problems still exist. For example, in the process of leaf identification based on image analysis, the feature of extraction was usually determined by manual analysis. The differences among plant species were not solved, and the differences among plant datasets with the same features would be produced. In the case of complicated background shooting, the identification accuracy of conventional approaches reduced significantly. Furthermore, traditional leaf identification also has some weaknesses that the training image dataset contains too little information about the plants and lacks of complex backgrounds [36]. To this end, we have created a dataset of 14 different trees that contain more complex information about the entire plant.

Overall, most of the related works used merely leaf images with simple, light and untextured background for plant species recognition. Therefore, we aim to recognize tree species by the whole plant images, which not only increase the recognition accuracy, but may enable remote recognition in the natural environment. Although these applications are promising, their performances are still far from the requirements of a real-world ecological in-field scenario [39]. Making accurate plant observations from the mass of users requires to equip them with much more accurate identification tools [78].

## 2. Method

### 2.1. Dataset

We created an image dataset of 14 tree species of the most common and endemic in Taiwan, including *Koelreuteria henryi*, *Liquidambar formosana*, *Ficus microcarpa*, *Terminalia catappa*, *Cinnamomum camphora*, *Delonix regia*, *Alstonia scholaris*, *Roystonea regia*, *Cassia fistula*, *Bischofia javanica*, *Melia azedarach*, *Melaleuca leucadendra*, *Terminalia mantaly*, and *Millettia pinnata* [79,80,81]. The raw database contained more than 30,000 photos. After deleting unqualified pictures with redundant, interfering backgrounds and non-targets, the dataset consisted of 2332 field images taken by mobile devices with natural backgrounds, different resolutions, and a wealth of information on the entire tree including leaves, branches, stems, and so on as a whole. It featured visually similar species, captured in a wide variety of situations. The training set contained 1843 images, and the test set included 489 images. A view of the image distribution per selected class was shown in Table 3.

### 2.2. Implementation and Preprocess

The model implementation was based on the open source deep learning framework keras [82]. The algorithm was implemented using Python 3.6.8 (Python Software Foundation, Delaware, USA) on Tensorflow 1.13 (Google LLC, Mountain View, CA, USA) [83], the libraries keras 2.3.0. (Google LLC, Mountain View, CA, USA), and run on Windows 10 (Microsoft Corporation, Redmond, WA, USA) with a NVIDIA GeForce 1070 GPU (NVIDIA Corporation, Santa Clara, CA, USA) for deep learning. Image preprocessing included transforming each image to gray-scale, then applying Gaussian filters, and lastly thresholding. All input image size in our CNN backbone model was resized to 448 × 448 pixels, and in our central attention model was cropped from the center, resized into 224 × 224 pixels. Each image was fed to the model as a full re-sized image and a center-cropped image, for the CNN backbone and central attention paths accordingly. Then, the per-pixel value was divided by 255, and all samples were shuffled for training [84].

We augmented the dataset using the following affine transformations to reach a dataset of 4000 samples. These transformations were label-preserving. The used transformations were random rotations of the image by an angle of up to 10 degrees; vertical or horizontal translation by a distance within 0.1 of the patch size randomly; horizontal flip: randomly flipping half of the images horizontally; and zooming inside pictures in the range 0.5–1.5 randomly.

### 2.3. Central Attention

**Figure 1:**
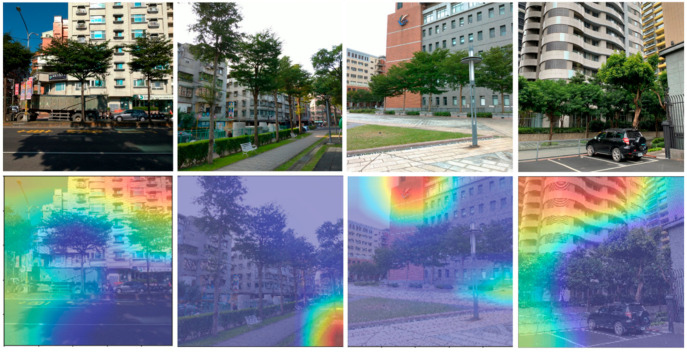
Grad-CAM visualizations. The top row is the original images in our tree species dataset. The bottom row is the Grad-CAM visualizations of our previous trained model. The heat maps localize class-discriminative regions, and red regions correspond to high score for class.

**Figure 2:**
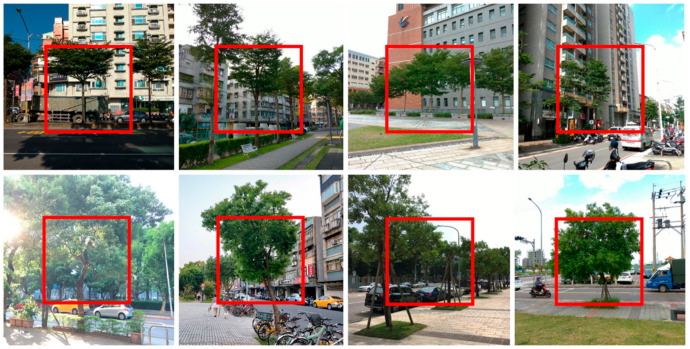
Samples of interfering backgrounds in the training dataset. Images in the upper row demonstrated redundant buildings and roads while in the lower row showed cars and sky as interfering background. The red boxes are the central attention part that would be cropped in the center as the input for central attention path in the dual path CNN.

As shown in Figure 1, interfering backgrounds, such as buildings, cars, street lights, pavement roads, and so on, could risk a mis-trained tree recognition model demonstrated by Gradient-weighted Class Activation Mapping (Grad-CAM) [82]. In addition, users tend to take photos with the subject in the center according to the observation and survey on user experiences. Therefore, in order to prevent the interference, we suggest focusing on the middle part of the full picture by cropping the center half of the image as illustrated in Figure 2.

### 2.4. Dual-Path CNN

**Figure 3:**
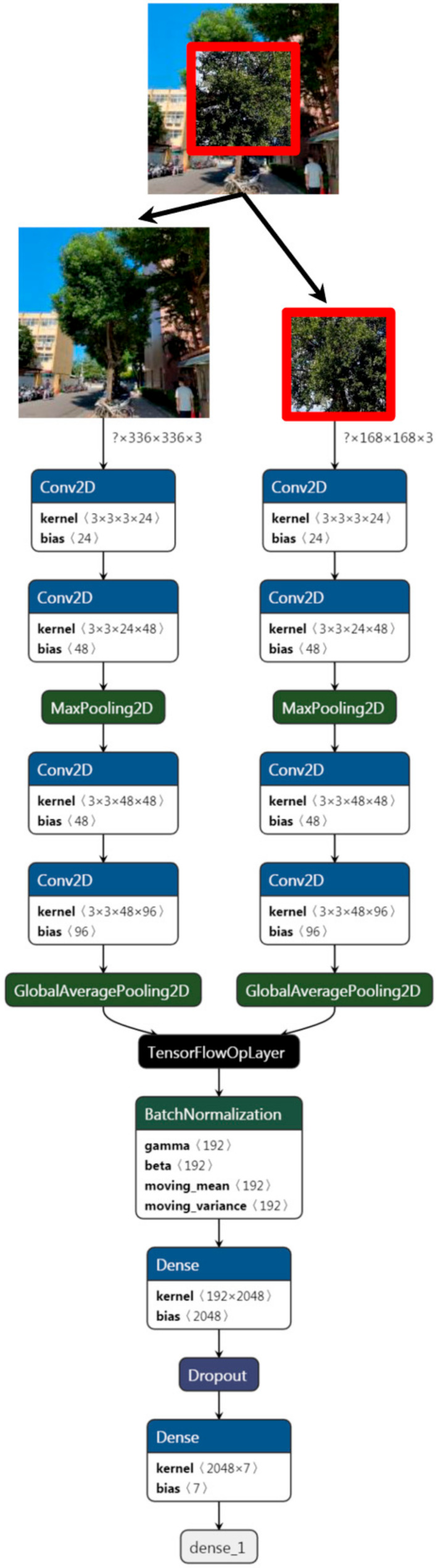
Architecture of the proposed dual path CNN framework. The left path is the backbone CNN architecture, and the right one is the central attention path with the central crop of the input image. Feature maps in the central attention path were concatenated with the corresponding backbone feature before class score computation.

We proposed a dual-path CNN architecture for tree species recognition. The architecture consisted of two sub-network pathways: (1) central attention with an InceptionV3 based pathway; and (2) an InceptionV3-based pathway. Figure 3 shows the architecture of our proposed network. Each of the two paths was independent from one another, but merged through concatenation of results, which were thus sent for classification. Feature extraction, hence comprised the classic CNN path, along with which was introduced the central attention concept, a simply visual attention focused on the central target.

To find the most appropriate backbone architecture, we tested the latest CNN-based models (data not shown). Then, InceptionV3 was selected as a backbone network to solve the plant recognition problem. The InceptionV3 was pre-trained in the ImageNet1000 dataset and received a red, green, blue (RGB) image with a size of 299 × 299 pixels and classified into its corresponding class. Inceptionv3 introduces inception modules. Inception modules help increase the width of the network. It is a convolutional block which is constituted by different kinds of convolutional kernels. Apart from 3 × 3 kernel is employed which is common used, other types such as 1 × 7, 7 × 7, 1 × 1, 1 × 3, and so on are also adopted for constructing networks. Large and small convolutional kernels are used together in one block. Big convolutional styles and feature maps are with little number of kernels, and small convolutional styles and feature maps are with large number of kernels [19]. This architecture consists of 159 layers including an alternating sequence of convolution (CONV), pooling layers and ends with a fully connected layer [85]. This architecture includes 23 million parameters. We used this model as a feature extractor to tree classification.

Additionally, the rectified linear unit, ReLu was used for the activation of each layer except for the last. A default learning rate of 0.01 was set. The Adam optimization function was used, while the categorical cross entropy was deployed as the loss function. We trained the data for 600 epochs and set the batch size to 50. We concatenated the results using the early fusion that performed better while integrating both networks and jointly training them end-to-end with fused representation linked directly to the species classes via a softmax layer that can be carried out before class score computation, such as during the feature learning stage, from which we adopted this method as described [9]. Afterwards, tuning the parameters of the network including activation function, mini batch size, epochs number, as well as the initial learning rate and study the accuracy and performance rate of different parameter variations of the CNN [86].

### 2.5. Object Detection

We employed YOLOv3 as the real-time object detection models. YOLOv3 introduced the multiscale windows generation to produce more accurate result and increased its speed substituting the backbone network with the Darknet-53 network, initialized from ImageNet [87].

The YOLOv3 model used in this work obtained the storage location of the training image and the marked pixel location of the target in the image and the class of the target by reading the txt text. This txt file was generated by reading a dataset using the VOC format. Therefore, the pictures collected in the scene were made into a dataset in the form of VOC by using an image annotation software called LabelImg [88]. It saves the object class information and position information marked in the image as a file in xml format for training. After using the rectangle to mark the target and select the target class, we save the file, and then the software generated the xml format label text with the same name as the image [89].

**Figure 4:**
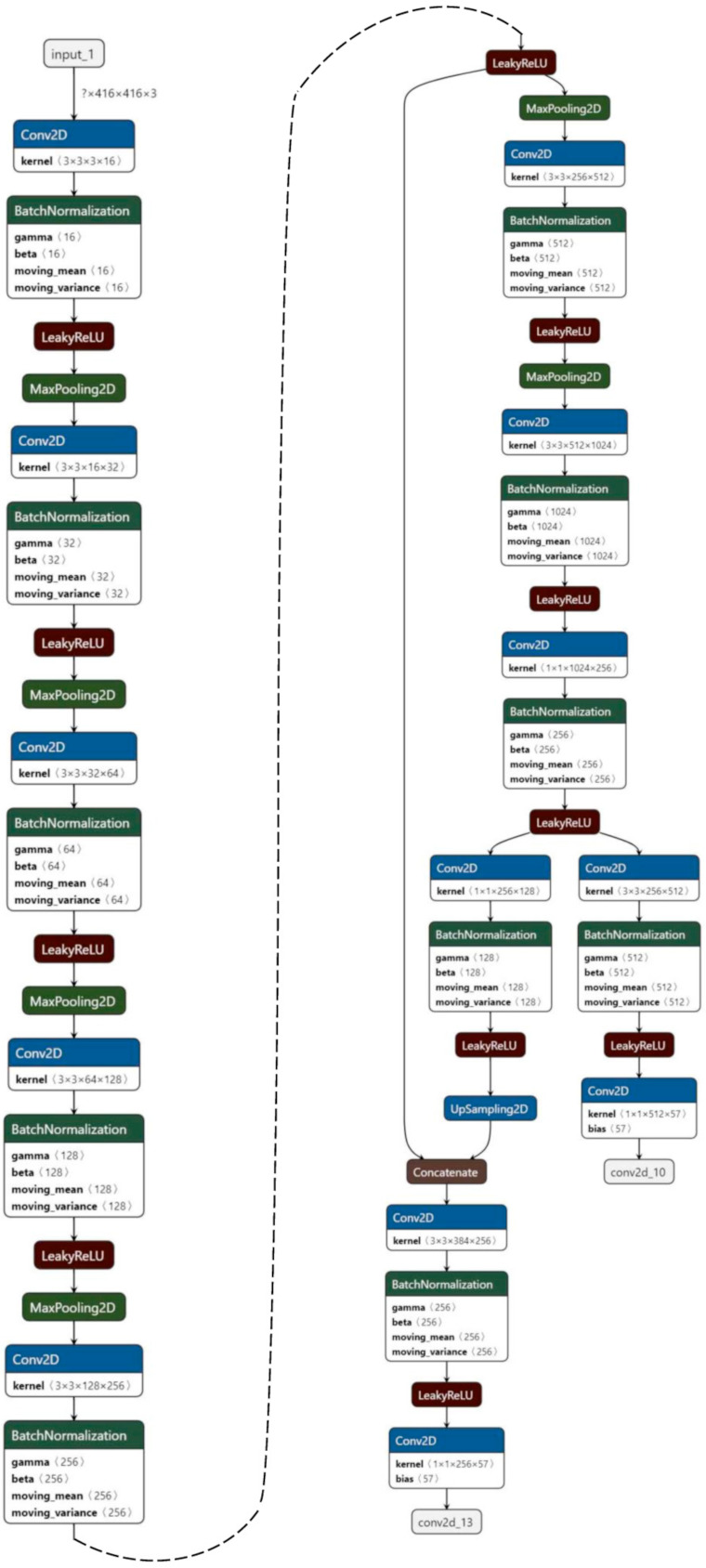
Object detection process flow diagram.

We also used intersection over union (IOU) and non-maximum suppression (NMS). In the training stage, we used the YOLOv3 provided code and default configuration [90,91]. The Adam optimization function was used. The learning rate was set to 0.00001. The value for both height and width was set to 416 × 416. The batch size is set to 3. Concerning the YOLOv3 parameters, the input image is subdivided to 8, 16, or 36 grids. Anchors that overlapped the ground truth object by less than a threshold value 0.5 were ignored (Figure 4).

### 2.6. Mobile Implementation

In our implementation, we provide interfaces to both an online and offline tree image database. The feature extraction could alternatively be performed at the server for more computationally expensive algorithms [40].

Here, we used the Tensorflow Lite framework to use in mobile environment. On Android, Tensorflow Lite leverages the Neural Networks API (NNAPI) to utilize all of the device hardware acceleration [92]. After the training was finished from a desktop environment, a Keras HDF5 model was built and then converted into a TensorFlow lite model to be embedded into the mobile environment for recognition. Based on the hardware capabilities of the device, it will efficiently distribute the workload of classification across available hardware, such as neural network hardware, GPUs, and digital signal processors (DSPs) [93].

In addition, according to the TensorFlow Lite documentation, although model quantization can lead to up to 0.8% decrease in accuracy, it may reduce the model size by four times (95.7 MB down to 23.9 MB) and the latency by 285 ms (1130 ms down to 845 ms) [94]. With the optimized storage size for InceptionV3 weights, this is well-suited for mobile applications.

## 3. Result

**Figure 5:**
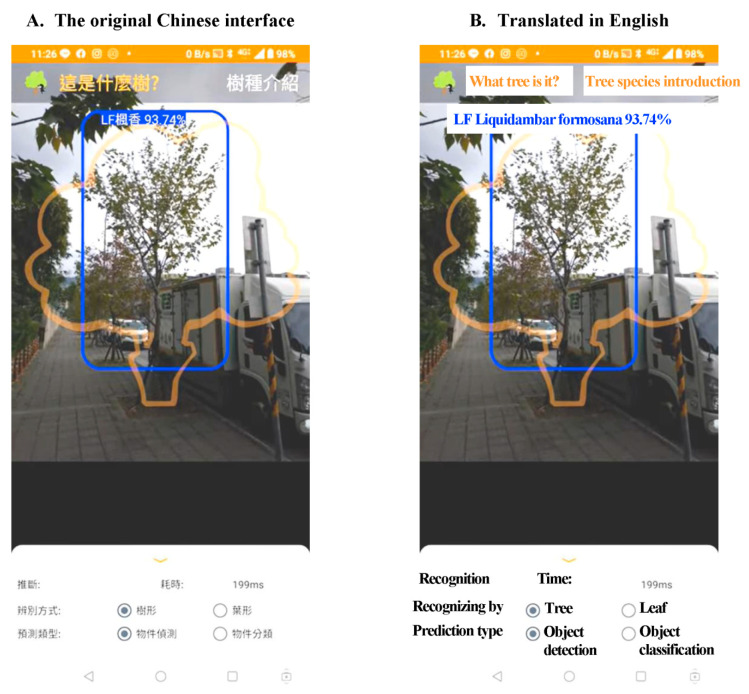
The screenshot of the developed mobile app. Users were instructed to place the object (tree) to be detected in the orange tree frame. In this detection, it took 199 mini seconds to frame the detected tree and show the result of *Liquidambar formosana* (LF). The user interface is shown in the original Chinese version (**A**), and translated in English (**B**).

The results demonstrate two key points. First, we build a tree species recognition system using a dual path CNN model constituted of a classic InceptionV3 CNN in combination with a central attention CNN. This method performs better than other existing methods in the same database. Second, we deliver this identification system for Android serving not only image recognition, but also real-time object detection functions (Figure 5).

### Central Attention and Dual-Path CNN

In order to evaluate the validity of the proposed framework, we use a couple of uni-path CNN networks to conduct the same experiment and compare the experimental results. The performance is measured by the average classification accuracy of different classes. The identification accuracy of the proposed dual path CNN is 77.1% while the uni-path classic InceptionV3 CNN is 74.1% and the uni-path central attention method is 75%.

**Figure 6:**
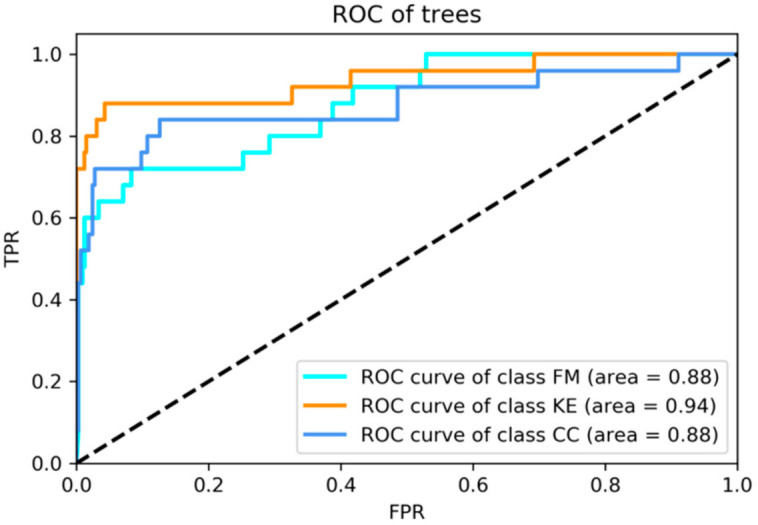
Receiver operating characteristic (ROC) curves of the top three major classes, *Ficus microcarpa* (FM), *Koelreuteria henryi* (KE), and *Cinnamomum camphora* (CC). TPR: true positive rate; FPR: false positive rate.

In addition, we have compared the proposed CNN method with InceptionResNetV2, which has been shown to have better learning performance than InceptionV3 [95], achieve at least 99.9% accuracy on the Middle European Woods dataset [96], and also win the LifeCLEF2018 challenge [97]. The experimental results were listed in Table 4. Values for the area under the receiver operating characteristic (ROC) curves (AUC), as well as true positive rate (TPR) in each class, are presented in Table 5. As shown in Table 4 and Table 5, most of the results in the proposed method for plant identification performed better than InceptionResNetV2. The possible reason may be that the amount of data collected in this study was scarce, so InceptionResNetV2, which has a more complex neural network structure, has a problem of overfitting. However, the proposed method obtains an average of 75.48% classification accuracy better than InceptionResNetV2 in the case of insufficient data, as shown in Table 4. Moreover, we used five-fold and 10-fold cross-validation to verify the effectiveness of the proposed method. Five-fold cross-validation represents that 80% of the original data is used as training data and 20% as test data. ROC curves of the top three major classes were shown in Figure 6.

**Figure 7:**
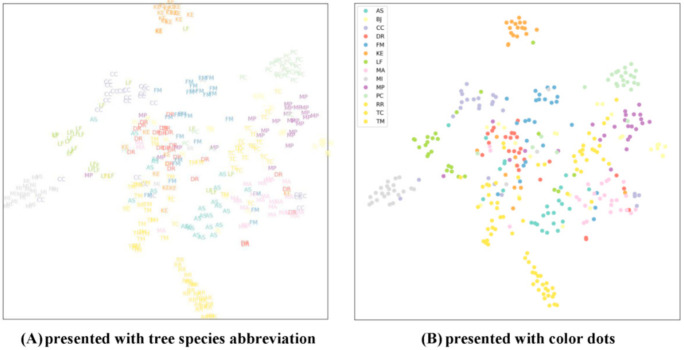
Two-dimensional T-distributed Stochastic Neighbor Embedding (T-SNE) visualization in each class of the 14 tree species is presented with tree species abbreviation (**A**) and with color dots (**B**). *Alstonia scholaris* (AS), *Bischofia javanica* (BJ), *Cinnamomum camphora* (CC), *Delonix regia* (DR), *Ficus microcarpa* (FM), *Koelreuteria henryi* (KE), *Liquidambar formosana* (LF), *Melia azedarach* (MA), *Melaleuca leucadendra* (MI), *Millettia pinnata* (MP), *Cassia fistula* (PC), *Roystonea regia* (RR), *Terminalia catappa* (TC), and *Terminalia mantaly* (TM).

The T-distributed Stochastic Neighbor Embedding (t-SNE) visualization method was used to project the generated multidimensional clusters into a two-dimensional graph. This methodology is capable of maintaining the cluster distances that are present in the high dimensional space when these are projected into the two-dimensional space, and thus, the differences among clusters can be visualized [98]. Figure 7 depicted the clusters generated by each class of the 14 tree species.

**Figure 8:**
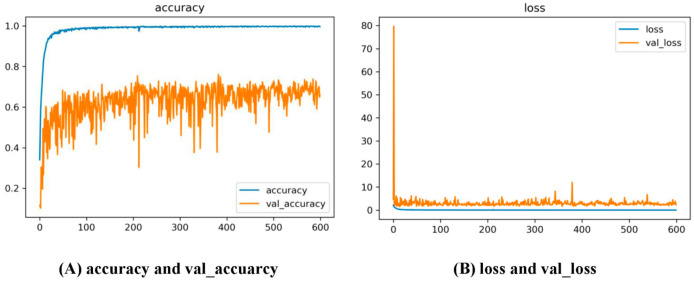
Training accuracy (**A**) and loss (**B**) curves of the proposed dual CNN method with epoch = 600 and five-fold cross-validation.

The training loss and accuracy curves of the proposed dual CNN method with epoch = 600 and five-fold cross-validation were shown in Figure 8.

**Figure 9:**
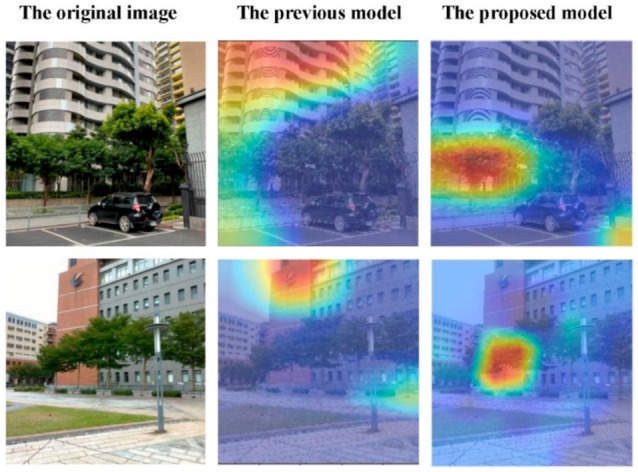
Grad-CAM visualizations of the proposed model. The left column is the original images in our tree species dataset. The middle column is the Grad-CAM visualizations of our previous trained model. The right column is the Grad-CAM results of our proposed model. The heat maps localize class-discriminative regions, and red regions correspond to high score for class.

The Grad-CAM results of the proposed method are shown in Figure 9.

## 4. Discussion

### 4.1. Central Attention and Dual-Path CNN

Although there are some tools already exist for plant recognition using a single organ (especially a leaf) with accuracies of 90% or more, we use the whole plant images to enable the remote recognition, which was one of the significances of this study. Moreover, the previous study showed that a plant classification method using a combination of leaf, flower and bark photos of the same tree species may achieve recognition accuracy on multi-organ better than the accuracy on a single organ [17]. In addition, previous studies reported 31.6% accuracy in the LifeCLEF2019 challenge [15], 69% top-5 identification rate in the Pl@ntNet application [4], and 67% accuracy in the iNaturalist application [44], which include the whole plant (long-range) images. In this study, the result of identification accuracy is 77.1% (Table 6), and has better learning performance (Figure 9).

By combining the two networks into a unified framework, the performance of tree species recognition can be improved. The finding is in line with previous studies discussed as follows. Shah et al. used a dual-path deep CNN to learn joint feature representations for leaf images, and optimize these features for the classification task based on marginalized shape context and shape-texture dual-path deep CNN [99]. The study showed that the dual-path CNN method outperformed other CNN methods, such as uni-patch CNN, texture-patch CNN, marginalized shape context with SVM classifier, multiscale distance matrix with SVM classifier, curvature histogram on Flavia, and other datasets [105].

Lee et al. further proposed a two stream convolutional neural network (TwoCNN) [9]. In TwoCNN, the two feature learning streams were trained on the whole and patch of images respectively. Although, TwoCNN can take discriminative information at different scales (both the whole and the patches of images), the training process required a more complex sample set that must provide both whole and segmented images for this network [106].

Rizk proposed a dual path, dual feature model for plant leaf identification [33]. The author used Sobel operators as primary and secondary vein extraction for vein patch generation, and then the dual-path CNN was employed for feature extraction. The first path was for leaf shape feature extraction while the second was for leaf venation feature extraction. The results showed an accuracy of 96.8 % tested on the Flavia dataset.

Dual-resolution dual-path CNNs, DualNets, were aimed to increase the object detection accuracy of small CNN models [34]. DualNets explicitly accepted dual inputs in different resolutions and extracted complementary visual features from these using dual CNN paths. The two paths in a DualNet were a backbone path (MobileNetV2) and an auxiliary path that accepted larger inputs and then rapidly down-sampled them to relatively small feature maps. Auxiliary features were extracted from the larger input with controllable computation, and then fused with the backbone features via a proposed progressive residual fusion strategy to enrich feature representation. This architecture, as the feature extractor, was further integrated with the Single Shot Detector to accomplish latency-sensitive visual object-detection tasks. The authors evaluated the resulting detection pipeline on Pascal VOC and MS COCO benchmarks. Results showed that the DualNets can improve the accuracy of those CNN detection applications sensitive to computation payloads.

A proposed dual-stream CNN, designed to perform robust indoor relocalization in challenging environments, took color images and depth images as the network inputs separately [35]. The network improved the relocalization accuracy by about 20% compared to the state-of-the-art deep learning method for pose regression, and also enhanced the system robustness in challenging scenes such as large-scale, dynamic, fast movement, and night-time environments.

Chen and Chiang suggested an auxiliary structure for deep model learning with insufficient data through additional alignment layers to transfer the weight of the auxiliary model to a new one [37]. Their results demonstrated that the auxiliary structure eliminated the overfitting problem and can improve the accuracy with only a few training samples. With the replacement of different auxiliary architecture, this method can be applied in different tasks, such like detection and retrieval.

Sun et al. [100] proposed the RTFNet utilizing RGB-Thermal fusion network for semantic segmentation of urban scenes for autonomous vehicle system. They used thermal images, and fuse both the RGB and thermal information in a novel deep neural network. The encoder-decoder design concept and ResNet were employed for feature extraction. A new decoder was developed to restore the feature map resolution. Their network achieved 63.1% accuracy in a public dataset [101]. Recently, they upgraded their model by employing DenseNet-161 as the backbone of the encoders. It generally consists of two encoders to extract features from input images and one decoder to restore the resolution. The two encoders take as input the three-channel RGB and one-channel thermal images, respectively. This upgraded model, FuseSeg-161, reached 70.6% accuracy in a public dataset [101], and 38.3% accuracy in the a RGB-Depth Scene Understanding Benchmark Suite (SUN-RGBD) v1 dataset [103], which outperformed the state of the art [102].

Wang et al. proposed a self-supervised approach to segment drivable areas and road anomalies for robotic wheelchairs. They firstly developed a pipeline named self-supervised label generator (SSLG) to automatically label drivable areas and road anomalies. Then, the segmentation labels generated by the SSLG were used to train several RGB-D data-based semantic segmentation neural networks. The accuracy was 75.57% in drivable area segmentation, and 88.19% in road anomaly segmentation of their self-constructed RGB-D dataset, which covers 30 common scenes where robotic wheelchairs usually work [104].

### 4.2. Challenges of Sampling in Current Automated Plant Species Identification

Inadequate benchmark database: Large-scale, well-annotated training datasets with representative data distribution characteristics are essential for the training of accurate and generalizable classifiers in Deep CNN [4]. Entire plant instead of individual organ: Multi-organ datasets performed remarkably well with an accuracy of 100% even if the size of the dataset was small [45]. Large intraspecific and small interspecific visual variation: Even professional botanists are challenged to properly distinguish species that can be identified only by almost invisible characteristics. Images of the same organ acquired from different perspectives often contain complementary visual information that could improve accuracy in observation-based identification using multiple images [4].

Therefore, we decided to collect our own database on the entire plant, and we have been very strict to select images to establish our dataset according to the principle of high variability. The original database contains more than 30,000 photos. After deleting unqualified pictures with redundant, interfering background and non-target, it turned out just 2000 more photos finally survived.

### 4.3. Solutions to Insufficient Data Quantity

To overcome data imbalance and overfitting, over-sampling or under-sampling can be used to adjust the class distribution from the dataset. Another simple way is to generate synthetic samples using algorithms like the Synthetic Minority Over-Sampling Technique by randomly sample the attributes from instances in the minority class. On the other hand, data augmentation can also help the network memorize the exact details of the training images via its options of resize, rotate and reflect images of the dataset [86].

In addition to increasing the data quantity by data augmentation, we proposed to improve data quality by focusing on the central attention to diminish distracting backgrounds in the natural environment. Specifically, we create an independent path with central attention images as inputs for deep learning, and finally fusion it with the other classic CNN path to complement the traditional deep learning network.

### 4.4. Transfer Learning

Transfer learning was adopted because it is much faster and easier to fine-tune a network than to train from scratch. Additionally, the pre-trained network has already learned a rich set of features which may be applied to a wider range of tasks [86].

Wick and Puppe proposed a pre-trained weight on the Caltech-256 dataset [107] that differed substantially from leaf classifications, and a nine-layer CNN, by which almost perfect accuracy were achieved on the Flavia and Foliage dataset [108]. Reyes et al. pre-trained the proposed model with seven layers CNN and additional prediction layer added at the top using the ILSVRC2012 dataset of 1.8 million images, including branch, flower, fruit, leaf, stem, etc., as well as scans [109]. They used a fine-tuning end-to-end strategy without hand-engineered components to transfer learned recognition capabilities from general domains, and implemented this model on the pl@ntView dataset. The results showed that the model can classify images of flowers and leaf scans with higher accuracy than the rest of views. The model obtained an average precision of 48.6% when identifying single images in the test set. Toma et al. studied a transfer learning approach on the PlantCLEF2017 challenge for automatic plant image classification in order to evaluate the performance of a system built with noisy data against one built using trusted data. The proposed method was based on the AlexNet CNN model, and fine-tuned using PlantCLEF Encyclopedia of Life training data including 10,000 species with about 260,000 plant images. They achieved 47.03% accuracy, and ranked within top 10 in the competition [110]. Van Horn et al. used multiple pre-trained CNN models including Inception ResNet V2 Squeeze-and-Excitation (SE), Inception ResNet V2, Inception V3 SE, ResNet152 dropout layer (drp), ResNet101 drp, ResNet152, ResNet101, and MobileNet. The best validation accuracy they obtained was 67.3% top-1 accuracy and 87.1% top-5 accuracy on the public dataset, iNaturalist Classification and Detection Dataset, using the Inception ResNet V2 SE model [44].

This present method allows us to use the existing model with a small amount of data and training time to achieve good results that is more useful for simple classification problems with a small dataset. Besides, the parameters of this network have been reduced that break down the training difficulty. Notably, if we update the pre-training parameters of all the layers, the identification result will improve. The benefit of fine-tuning without completely retraining the model increases efficiency, as the accuracy of new training models generally increases slowly from very low values, but fine-tuning enables to get a better result with less iteration [36]. Transfer learning is efficient by using a pre-trained deep representation as a source architecture to create a new architecture [111].

Interestingly, it can use any pre-trained networks to train the new deep model given insufficient training samples or a lack of the initial weights. Each path in the dual-path deep neural network is flexible, and we can choose the different deep models according to different tasks requirement [37].

Finally, we believe further fine-tuning of the CNN parameters could enhance the overall performance and accuracy of the results. Further experiments with the model’s weights and layer count could achieve a higher accuracy more efficiently. In addition, enlarging the database through merging with other image banks could also improve the learning process in the CNN [33].

## 5. Conclusions

To begin with, we propose a central attention concept based on user experience analysis and previous studies. The concept helps focus on the target instead of backgrounds in the image, and could prevent model training from confused vision for tree species recognition. Next, a dual-path CNN model is developed where the two different sub-networks are independent and accept individual input of either an original image or a central cropped one, respectively. A concatenation layer is then used to fuse the output of two independent sub-networks. Furthermore, we create a plant dataset of 14 species of the most common trees in Taiwan for model training and validation, respectively.

The experimental results demonstrated that the central attention combined with a dual path CNN in a whole plant recognition can achieve very competitive performance on an accuracy rate 77.1% compared to the existing deep learning algorithm, 31.6 % accuracy in the LifeCLEF2019 challenge [15], 69% top-5 identification rate in the Pl@ntNet application [4], and 67% accuracy in the iNaturalist application [44]. Finally, we implemented the recognition model into a prototype system of an online/offline available tree species identification working on a consumer mobile platform that can not only identify the tree species by image recognition, but also real-time detect and classify for live streaming camera.

However, there are still several issues to be addressed in the future. The image data were collected from September to December in 2019. A limitation of this study is that the query image is restricted to plant species of the common roadside trees in Taiwan during fall and winter. In addition, the provided dataset, which contains only 14 species and 2332 images, would have limited field impact. However, these Taiwan-endemic roadside trees have regional specificity (e.g., *Koelreuteria henryi* [112], *Liquidambar formosana* [113]), and are possibly absent in other existing datasets. Moreover, although many datasets with thousands of species and millions of images already exist, most of them present a single organ, such as a leaf, per image. The provided dataset in this study consists of whole plant (long-range) images, facilitating not only a wealth of information on the entire tree but remote detection.

In the future we plan to extend the dataset to include more variant classes in different seasons to cover more plant species and growing stages. We also plan on trying different pre-trained CNN models to study accuracy and performance of the models. Furthermore, we plan to transfer this proposed model of image recognition to other areas such as health promotion. It has been suggested that a heterogeneous transfer learning framework may extend the transfer learning from one image classification dataset to another [114,115,116]. The present tree recognition model may serve as a CNN framework to apply for the health promotion area, such as automatic detection of human postures, medicine, etc., by deep transfer learning.

## Figures and Tables

**Table 1:** Summary of related works in deep leaning.

Authors, Year	Image Type	Dataset	Method	Accuracy
Lee et al., 2015, 2017 [9,46]	Leaf	MalayaKew Leaf dataset	Convolutional Neural Network (CNN) + AlexNet	99.7%
Chopra, 2015 [47]	Leaf	TreeID	Three-layer CNN	75%
Wu et al., 2016 [48]	Leaf	ICL	Four-layer CNN + PReLU activation function	94.8%
Bao et al., 2019 [49]	Leaf	Flavia and Swedish leaf dataset	Histogram of oriented gradients (HOG) + support vector machine (SVM) classifier + five-layer CNN	95.6%
Zhang et al., 2015 [50]	Leaf	Flavia	Seven-layer CNN	94.69%
Barre et al., 2017 [51]	Leaf	LeafSnap, Flavia, and Foliage datasets	17-layer CNN	97.9%
Zhu et al., 2018 [45]	Leaf, flower, fruit, branch, stem	LifeCLEF2015 dataset	19-layers CNN + SVM	Leaf (67.10%), flower (88.80%), fruit (90.20%), branch (71.20%), stem (65.20%)
Pearline et al., 2019 [52]	Leaf	Leaf12 dataset	VGG 16 CNN architecture with logistic regression (LR)	97.14%
		Folio, Flavia, and Swedish leaf datasets	VGG 19 CNN architecture with LR classifier	Folio (96.53%), Flavia (96.25%) Swedish (99.41%)
Goëau et al., 2019 [15]	Plant	LifeCLEF2019 plant dataset	InceptionV4 and Inception-ResNet-v2 CNN	31.6%
Sun et al., 2017 [53]	Leaf	Flavia, BJFU100 datasets	26-layer ResNet	Flavia (99.65%), BJFU100 (91.78%)
Bodhwani et al., 2019 [1]	Leaf	LeafSnap	50-layer deep residual learning framework	93.09%

**Table 2:** Summary of related works in mobile applications.

Authors, Year	Image Type	Mobile Applications	Method	Accuracy
Belhumeur et al., 2008 [56]	Leaf	LeafView	Nearest neighbor (NN) classifier	Top ten matches 90–97%
Kumar et al., [42]	Leaf	Leafsnap	NN	73%
Goëau et al., 2013 [43]	Leaf, flower, fruit and bark	Pl@ntNet	Approximate k-NN search + administration of material purchases -local sensitive hashing (AMP-LSH)	69% top-5 identification rate
Cerutti et al., 2013 [57]	Leaf	Not available (N/A)	Linear regression (LR) + adaptive local mean (AM)	N/A
Ma et al., 2013 [58]	Leaf	ApLeafis	Content-based image retrieval (CBIR) HSV (Hue, Saturation, Value), Wavelet, pyramid histogram of orientated gradients (Phog) + tophat + cropped	90%
Nguyen et al., 2013 [59]	Leaf	N/A	Speeded Up Robust Features (SURF) + Bag of Words (BOW) + support vector machine (SVM)	95.94% [60]
Prasad et al., 2013 [61].	Leaf	N/A	k-NN classifier	70.09–91.34%
Wang et al., 2013 [40].	Leaf	N/A	Multiscale shape descriptor based on the concave and convex measures + k-NN	86.86–96.05%
Priyankara et al., 2015 [62]	Leaf	N/A	Scale Invariant Feature Transform (SIFT) + BOW feature vector + SVM classifier.	96.48%
Zhao et al., 2015 [63]	Leaf	ApLeaf	HSV, Wavelet, Phog + tophat + cropped	90%
Van Horn et al., 2017 [44]	Plant and animal	iNaturalist	Inception ResNetV2 + Squeeze-and-Excitation (SE) blocks	67.3%

**Table 3:** Image count per class in the 14 tree species dataset.

Class Label	Image Count
*Koelreuteria henryi*	203
*Liquidambar formosana*	150
*Ficus microcarpa*	295
*Terminalia catappa*	62
*Cinnamomum camphora*	201
*Delonix regia*	105
*Alstonia scholaris*	96
*Roystonea regia*	68
*Cassia fistula*	105
*Bischofia javanica*	164
*Melia azedarach*	56
*Melaleuca leucadendra*	100
*Terminalia mantaly*	170
*Millettia pinnata*	68
Total image count	1843

**Table 4:** Comparison of accuracy between the proposed method and InceptionResNetV2.

K-Fold	5-Fold (Training Data Size = 1754, Testing Data Size = 439)		10-Fold (Training Data Size = 1973, Testing Data Size = 220)	
Method	InceptionResNetV2	The Proposed Method	InceptionResNetV2	The Proposed Method
Accuracy	56.95	69.02	71.36	75.48

**Table 5:** Comparison of true positive rate (TPR) and area under the curve (AUC) between the proposed method and InceptionResNetV2 in each class.

K-Fold	5-Fold (Training Data Size = 1754, Testing Data Size = 439)				10-Fold (Training Data Size = 1843, Testing Data Size = 350)			
Method	InceptionResNetV2		The Proposed Method		InceptionResNetV2		The Proposed Method	
Class	TPR	AUC	TPR	AUC	TPR	AUC	TPR	AUC
AS	0.7297	0.8201	0.8919	0.8726	0.7500	0.8275	0.7500	0.8325
BJ	0.7083	0.7813	0.6875	0.7760	0.7500	0.8112	0.6250	0.7513
CC	0.4500	0.6962	0.4250	0.6849	0.4615	0.6870	0.6154	0.7561
DR	0.6667	0.8020	0.6667	0.8092	0.2500	0.6178	0.4167	0.6867
FM	0.7302	0.7959	0.7302	0.8012	0.7857	0.8304	0.8571	0.8478
KE	0.6905	0.7986	0.7857	0.8412	0.8125	0.8523	0.8125	0.8695
LF	0.6061	0.7735	0.7576	0.8320	0.9231	0.9108	1.0000	0.9517
MA	0.6316	0.7515	0.1579	0.5730	0.5000	0.7360	0.1667	0.5740
MI	0.1923	0.5877	0.4231	0.6982	1.0000	0.9328	0.7368	0.8311
MP	0.5333	0.7478	0.6000	0.7800	0.8000	0.8762	0.7000	0.8262
PC	0.8148	0.8613	0.8889	0.9080	1.0000	0.9539	0.9286	0.9182
RR	1.0000	0.9752	0.8750	0.9210	0.7500	0.8608	1.0000	0.9764
TC	0.3571	0.6715	0.9286	0.9431	0.7500	0.8608	1.0000	0.9764
TM	0.8286	0.8623	0.9143	0.8792	0.8125	0.8695	0.9375	0.9295
Average	0.6385	0.7804	0.6952	0.8085	0.7390	0.8305	0.7533	0.8377

Note: *Alstonia scholaris* (AS), *Bischofia javanica* (BJ), *Cinnamomum camphora* (CC), *Delonix regia* (DR), *Ficus microcarpa* (FM), *Koelreuteria henryi* (KE), *Liquidambar formosana* (LF), *Melia azedarach* (MA), *Melaleuca leucadendra* (MI), *Millettia pinnata* (MP), *Cassia fistula* (PC), *Roystonea regia* (RR), *Terminalia catappa* (TC), *Terminalia mantaly* (TM).

**Table 6:** Comparison of previous dual-path CNN works and our proposed method.

Authors, Year	Image Type	Dataset	Method	Accuracy
Shah et al., 2017 [99]	Leaf	Flavia, Leafsnap, ImageClef	Dual-path deep convolutional neural network (CNN) Path 1: marginalized shape context Path 2: shape + texture	Flavia (99.28%), Leafsnap (95.61%), ImageClef (96.42%)
Lee et al., 2017 [9]	Leaf	MalayaKew Leaf dataset	Dual-path CNN Path 1: CNN Path 2: AlexNet	99.7%
Rizk, 2019 [33]	Leaf	Flavia	Dual-path CNN Path 1: leaf shape feature extraction Path 2: leaf venation feature extraction	96.8%
Pan et al., 2019 [34]	Pascal VOC: person, animal, vehicle, indoor (20 object classes) MS COCO: animal, vehicle, furniture, etc (91 classes)	Pascal VOC and MS COCO dataset	Dual-resolution/input dual-path CNNs (DualNets) Path 1: backbone path MobileNetV2 with 300 pixels input Path 2: auxiliary path with larger inputs (600 pixels) but less stacked layers Feature maps in the auxiliary path are fused with the corresponding backbone feature in a residual-learning manner as long as their dimensions meet.	70.4%
Li et al., 2018 [35]	Fire, heads, chess, pumpkin, office, redktichen, and stairs	Microsoft 7-Scenes dataset	Dual-path CNN Path 1: color image inputs Path 2: depth image input	Relocalization accuracy improved by about 20% compared with the state-of-the-art deep learning method for pose regression
Chen and Chiang, 2018 [37]	Human actions People Playing Musical Instrument (PPMI): human interaction, musical instruments (48 classes) Willow: action (7 classes) Uiuc-sports: action (8 classes)	PPMI dataset, Willow dataset, Uiuc-sports dataset	Dual-path CNN Path 1: CNN Path 2: CNN pre-trained on a large dataset, a large generative deep learning model (e.g., AlexNet, GoogleNet)	PPMI (42.7%), Willow (44.8%), Uiuc-sports (81.6%)
Sun et al., 2019 [100]	Car, person, bike, curve, car stop, guardrail, color cone, bump	A public dataset [101]	Encoder-Decoder design concept ResNet RTFNet: RGB-Thermal Fusion Network. Fusing both the RGB and thermal information in a novel deep neural network	63.1%
Sun et al., 2020 [102]	Car, person, bike, curve, car stop, guardrail, color cone, bump SUN-RGBD v1: room scenes, furniture, etc.	A public dataset [101], SUN-RGBD v1 dataset [103]	FuseSeg: RGB and thermal data fusion, generally consisting of two encoders to extract features from input images and one decoder to restore the resolution. The two encoders take as input the three-channel RGB and one-channel thermal images, respectively. DenseNet-161	A public dataset (70.6%) SUN-RGBD v1 (38.3%)
Wang et al., 2019 [104]	30 common scenes where robotic wheelchairs usually work (e.g., sidewalks and squares) 18 different kinds of road anomalies that robotic wheelchairs may encounter in real environments	Ground mobile robots perception dataset (RGB-D dataset)	Self-Supervised Label Generator (SSLG) + RGB-D data-based semantic segmentation neural networks	75.57~88.19%
Our proposed method	Whole plant (long-range)	Self-collected dataset: 14 species of the most common and endemic trees in Taiwan	Dual-path CNN Path 1: InceptionV3 with original image inputs Path 2: InceptionV3 with central cropped image inputs	77.1%

## Data Availability

Data sharing is not applicable to this article.
